# Effect of One-Week Salt Restriction on Blood Pressure Variability in Hypertensive Patients with Type 2 Diabetes

**DOI:** 10.1371/journal.pone.0144921

**Published:** 2016-01-05

**Authors:** Hiroyuki Iuchi, Masaya Sakamoto, Hirofumi Suzuki, Yosuke Kayama, Kennosuke Ohashi, Takeshi Hayashi, Sho Ishizawa, Tamotsu Yokota, Katsuyoshi Tojo, Michihiro Yoshimura, Kazunori Utsunomiya

**Affiliations:** 1 Division of Diabetes, Metabolism and Endocrinology, Department of Internal Medicine, Jikei University School of Medicine, Tokyo, Japan; 2 Department of Cardiology, Jikei University School of Medicine, Tokyo, Japan; Tokai University, JAPAN

## Abstract

**Background:**

Increased short-term blood pressure (BP) variability on 24-hour ambulatory BP monitoring (ABPM) is known to be a risk factor for cardiovascular events. However, very few studies have evaluated the effect of salt restriction on BP variability particularly in hypertensive patients with type 2 diabetes. This study aimed to investigate the effect of salt restriction on systolic BP (SBP) variability.

**Methods and Results:**

10 hypertensive patients with type 2 diabetes and not receiving antihypertensive agents were enrolled in the study. After admission, all patients received a salt-restricted diet and appropriate anti-diabetic treatments and were followed up for 7 consecutive days using ABPM. After the 7-day treatment, the median [interquartile range (IQR)] coefficient of variation (CV) for diurnal SBP variability changed from day 1 to day 7–13.0 [10.8 to 16.8] % to 13.3 [9.1 to 18.9] % (*P* = 0.959)—and the median [IQR] change between days 1 and 7 was -0.3 [-3.2 to 2.9] %. In addition, CV for BP variability and circadian rhythm of BP varied greatly on a day-by-day basis for 7 days, compared to mean BP values. Interestingly, increased SBP variability was associated with greater day-by-day changes in circadian rhythm of BP.

**Conclusions:**

Salt restriction during 7-day hospitalization led to a -0.3 [-3.2 to 2.9] (median [IQR]) % change from baseline in CV for diurnal SBP variability in 10 hypertensive patients with type 2 diabetes not receiving antihypertensive agents.

**Trial Registration:**

UMIN Clinical Trials Registry UMIN000016243

## Introduction

Hypertension and diabetes mellitus are highly likely to coexist and hypertension in diabetic patients constitutes a major risk factor for cardiovascular disease (CVD) [1]. In recent years visit-to-visit blood pressure (BP) variability has been shown to increase cardiovascular events independently of mean BP values [2]. Furthermore, in patients with diabetes, visit-to-visit BP variability not only increases cardiovascular events, but it is also correlated with the progression of micro- and macro-vascular complications of diabetes, thus suggesting that BP variability is an important risk factor as shown by the ADVANCE study [3].

While visit-to-visit BP variability is regarded as a measure of long-term BP variability, 24-hour ambulatory blood pressure monitoring (ABPM),as recommended by the AHA for evaluation and management of hypertension, is also a useful measure of short-term BP variability in individual patient. In addition, increased short-term BP variability, as shown by the standard deviation (SD) of diurnal and nocturnal BP from ABPM, has also been shown to be correlated with CVD [4,5]. This observation points to the need to control, not only visit-to-visit BP, but also short-term BP variability. Moreover, diabetic patients were reported to have association not only with increased short-term BP variability but also with impaired circadian rhythm of BP, as calculated by the degree of the nocturnal BP fall compared to diurnal BP. Therefore, given that there are at least two risk factors covered by the indices for 1-day BP derived from ABPM, treatment needs to be reduce short-term BP variability but also correct the circadian rhythm of BP.

It was reported that an angiotensin II receptor blocker (ARB) given for 3 months led to a significant decrease in mean BP value as well as in short-term BP variability in hypertensive patients [6]. It is thus generally assumed that decreases in BP values go hand in hand with those in BPV in ABPM-based intervention studies of antihypertensive agents.

While salt restriction is generally reported to be crucial for BP control, it is also useful for reducing cardiovascular events in diabetic patients [7]. Furthermore, a previous study showed that one week of salt restriction decreases mean BP values in comparison with a high salt diet [8]. However, very few studies have investigated the effect of lifestyle modification with salt restriction on BP variability. In particular, no previous reports have investigated the effect of salt restriction on short-term BP variability.

In addition, as sufficient salt restriction is not widely practiced in Japan, salt intake among the population is shown to be greater than that in the rest of the world [9] and still remains high at about 10 g/day. This appears to account in part for a higher incidence of cerebral infarction in Japan than that in other countries, suggesting a compelling need to optimize salt intake among the population as soon as possible [10]. Therefore, it is of particular interest to the Japanese population associated with excessive salt intake to investigate the effect of salt restriction on BP variability.

The purpose of this study, therefore, was to evaluate the effect of salt restriction in an inpatient setting on short-term BP variability using ABPM and continuous glucose monitoring (CGM) in the 7-day ABPM and CGM Study (7DACS).

## Methods

### Study design

The 7DACS was designed and conducted as a prospective intervention study. From the day of their admission onwards, all participants were subjected to salt restriction (less than 6 g/day) as well as caloric restriction (25–30 kcal/kg) and received standard diabetes education and treatment with insulin or oral anti-diabetic agents. Participants were instructed to limit their exercise to walking of short duration. No antihypertensive agents were allowed during the study. ABPM- and CGM-derived parameters were measured, urine collected, casual urine samples taken, and body weight measured for 7consecutive days in the participants, while blood samples were taken on the day after admission.

### Ethics statement

The study protocol was approved by the Ethics Committee of Jikei University School of Medicine on October 24, 2013. All participants gave written informed consent to participate. Participants were recruited from October 24, 2013, and the last participant was followed up until May 17, 2014. The study was registered with UMIN with the identifier UMIN000016243. As all of the participants received the regular inpatient treatment and were not allocated, there were no treatment differences among the participants. For this reason, trial registration was done post-recruitment to allow for tracking of study results. The authors confirm that all ongoing and related trials for this drug/intervention are registered.

### Participants

Hypertensive patients with type 2 diabetes being treated as outpatients at Katsushika Medical Center, Jikei University School of Medicine, Tokyo,and not receiving antihypertensive agents, were enrolled in this prospective intervention study.

Type 2 diabetes mellitus was defined according to the ADA criteria [11]. Hypertension was defined as systolic BP (SBP) 140 mmHg or greater, or diastolic BP (DBP) 90 mmHg or greater, based on at least 2 BP measurements.

Participants were excluded if they had been diagnosed with type 1 diabetes, secondary diabetes, diabetic ketoacidosis, advanced dehydration, advanced renal impairment, advanced hepatic impairment, evidence of CVD, malignancy, or if they were pregnant.

### Blood pressure measurement and definition of blood pressure variability

Immediately after admission, all participants were equipped with the ABPM device (TM2425; A&D, Tokyo, Japan) and BP was measured in the left upper extremity at 30-minute intervals between 06:00 and 22:00 and at 1-hour intervals between 22:00 and 06:00 for 7 consecutive days (days 1 to 7), with day 1 defined as 24 hours immediately after admission.

Diurnal BP and nocturnal BP were defined as BP values measured between 09:00 and 21:00 (diurnal) and between 00:00 and 06:00 (nocturnal), respectively, with measured values taken from at least 70% of valid readings being used for analyses. Mean BP values (mmHg) and coefficients of variation (CV) (SD*100/mean, %) were calculated for diurnal and nocturnal BPs. Circadian rhythm of BP was calculated as nocturnal systolic BP (SBP)/diurnal SBP, and extreme dippers, dippers, non-dippers and risers were defined as nocturnal/diurnal SBP ratios ≤ 0.8, 0.8 to ≤ 0.9, 0.9 to ≤ 1.0, and > 1.0, respectively [12].

### Urine collection and body weight measurement

Casual urine sampling and 24-hour urine collection were performed daily. Urine collection was re-initiated for the following 24 hours, and this process was repeated until the end of day 7. Estimated sodium intake was calculated based on casual urine samples [13]. Urinary C-peptide and urinary albumin were calculated based on the urine collected on day 1. Body weight was measured before breakfast each day.

### Glycemic variability assessment

Immediately after admission, all participants were equipped with the CGM device (iPro2; Medtronic MiniMed, USA), had their glucose levels measured for calibration purposes 1 hour after admission, and had their glucose measured continuously for 7 days.

### Measurement of the ratio of low- to high-frequency components

The ratio of low- to high-frequency power (LF/HF ratio) was used as a measure of sympathetic and parasympathetic activity and measured using a Polar heart rate monitor (RS800CX, Polar, Finland), with its sensor attached to the chest and its watch-like recorder worn around the wrist over 24 hours on the fourth day after admission.

### Laboratory analysis

Blood samples were taken from all participants before breakfast on the morning of the day after admission. Plasma glucose, HbA1c, LDL-cholesterol, HDL-cholesterol, triglyceride, creatinine, and plasma C-peptide were measured according to the usual methods.

### Statistical analysis

We hypothesized that one-week salt restriction could result in decreases in diurnal mean SBP of about 10 to 20 mmHg, as well as in a 2% decrease in CV for diurnal SBP variability which was comparable to that with antihypertensive therapy [6], in diabetic patients placed under inpatient conditions in which their diet, exercise and sleep duration. Due to concerns about tolerability of undergoing both ABPM and CGM for 7 consecutive days, the number of patients was set 10 in this study.

There was no obvious consensus on whether diurnal or nocturnal SBP variability was more important. Diurnal BP had more number of measurements than nocturnal BP in this study. For that reason, we considered that the primary endpoint was change in CV for diurnal SBP variability.

Data were expressed as medians (interquartile range, 25% to 75%). Two-group comparisons between days 1 and 7 were performed using the Wilcoxon signed-rank test. Pearson’s correlation coefficients were used to examine correlations between 2 variables. A *P* value of < 0.05 was considered significant in all analyses. All statistical analyses were performed using SPSS Advanced Statistics version 22 (SPSS, Chicago, Illinois, USA).

## Results

### Characteristics of participants

**Fig 1:**
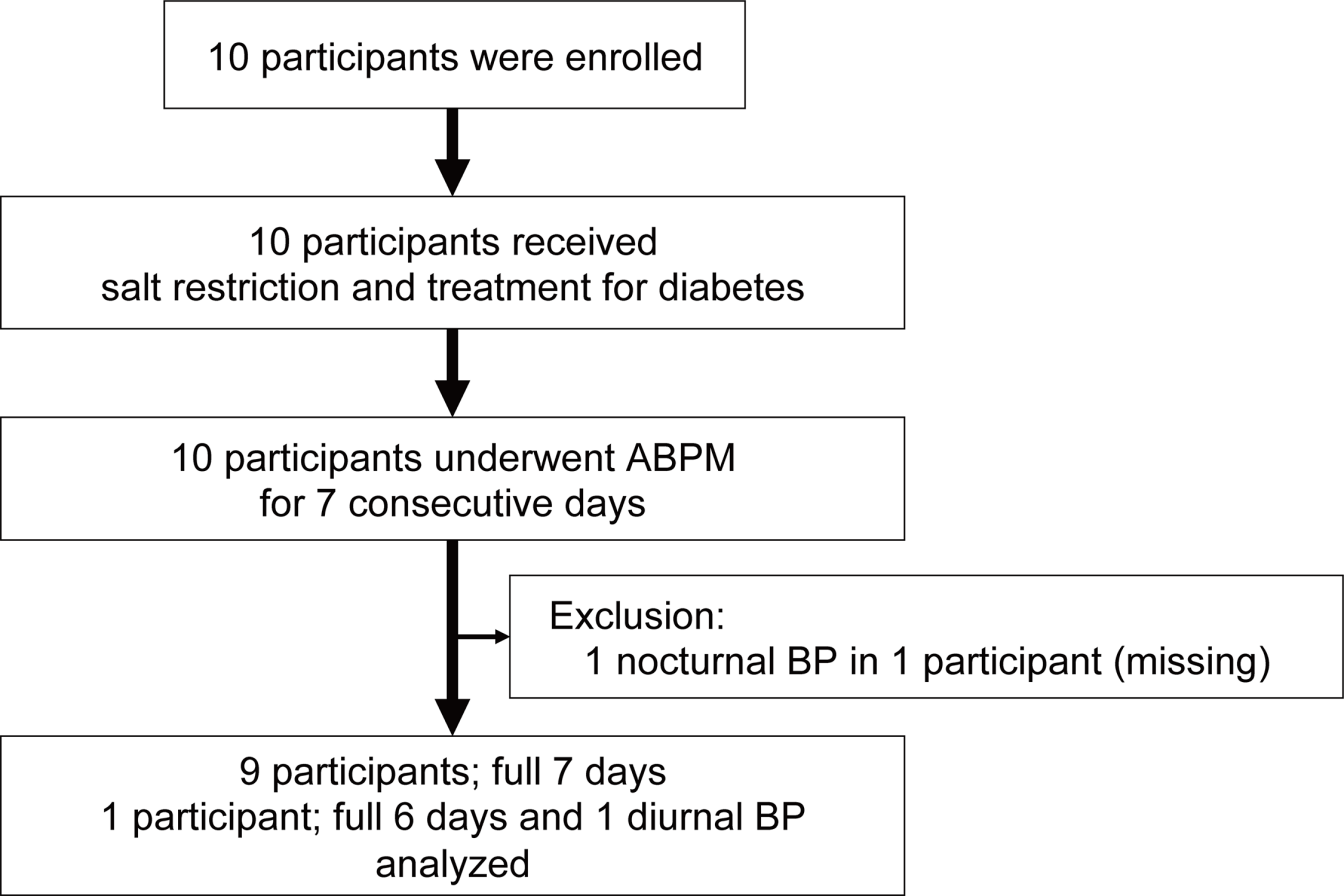
Flowchart of the study participants.

10 participants were enrolled in this study. A flow diagram of the participants’ enrollment and follow-up procedures is shown in Fig 1. The characteristics of the participants (baseline diabetic treatment, glucose control status, insulin-secretory capacity, and diabetic complications at admission) are summarized in Table 1.

**Table 1:** Baseline characteristics.

Variable	
N	10
Age (years)	60 (50 to 66)
Men (%)	70
Body Mass Index (day1)	26.3 (25.0 to 28.2)
Duration of diabetes (years)	2.0 (1.3 to 5.8)
Fasting plasma glucose (mg/dl)	162 (140 to 210)
HbA1c (%)	8.8 (7.9 to 10.9)
Urinary C peptide (μg/day)	76.0 (49.8 to 86.4)
Plasma C peptide (ng/ml)	1.4 (1.0 to 2.1)
C-peptide index	0.95 (0.90 to 1.03)
Triglyceride (mg/dl)	118 (104 to 146)
HDL cholesterol (mg/dl)	51 (47 to 56)
LDL cholesterol (mg/dl)	121 (109 to 128)
Creatinine (mg/dl)	0.61 (0.45 to 0.79)
eGFR (ml/min/1.73m^2^)	111 (75 to 117)
Urinary albumin excretion (mg/g·Cr)	19.5 (14.1 to 23.0)
Diabetic retinopathy (%)	30
CVR-R at rest (%)	2.8 (2.3 to 3.3)
Impaired vibration sensation in foot (%)	50
Orthostatic hypotension (%)	0
Diurnal LF/HF (%)	399 (249 to 474)
Nocturnal LF/HF (%)	153 (121 to 288)
CAVI	11.4 (10.0 to 14.2)
History of cardiovascular disease (%)	0
Treatment of diabetes	
Untreated (%)	20
Oral hypoglycemic agent (%)	50
Insulin (%)	30

Data are presented as medians (interquartile range: 25 to 75 percentiles).

eGFR, estimated glomerular filtration rate; CVR-R, coefficient of variation in the R-R intervals; LF/HF, low-frequency power / high-frequency power ratios; CAVI, cardio-ankle vascular index.

In about half of the participants, the estimated salt intake was shown to be higher than the mean salt intake in Japan and no participants had an estimated salt intake of less than 6.0 g on day 1. After the 7-day treatment, estimated sodium intake varied from day 1 to day 7; 9.8 (7.7 to 12.0) g/day to 6.8 (6.4 to 8.1) g/day, respectively. Furthermore, body mass index (BMI) also varied from 26.3 (25.0 to 28.3) kg/m^2^ to 25.6 (24.3 to 27.2) kg/m^2^.

There was a shift in the use of anti-diabetic agents among participants in favor of oral hypoglycemic agents over insulin (60% versus 40% of the treatments given). Of the CGM-derived parameters, glucose level was improved. There were no hypoglycemic events.

### Blood pressure parameters

**Fig 2:**
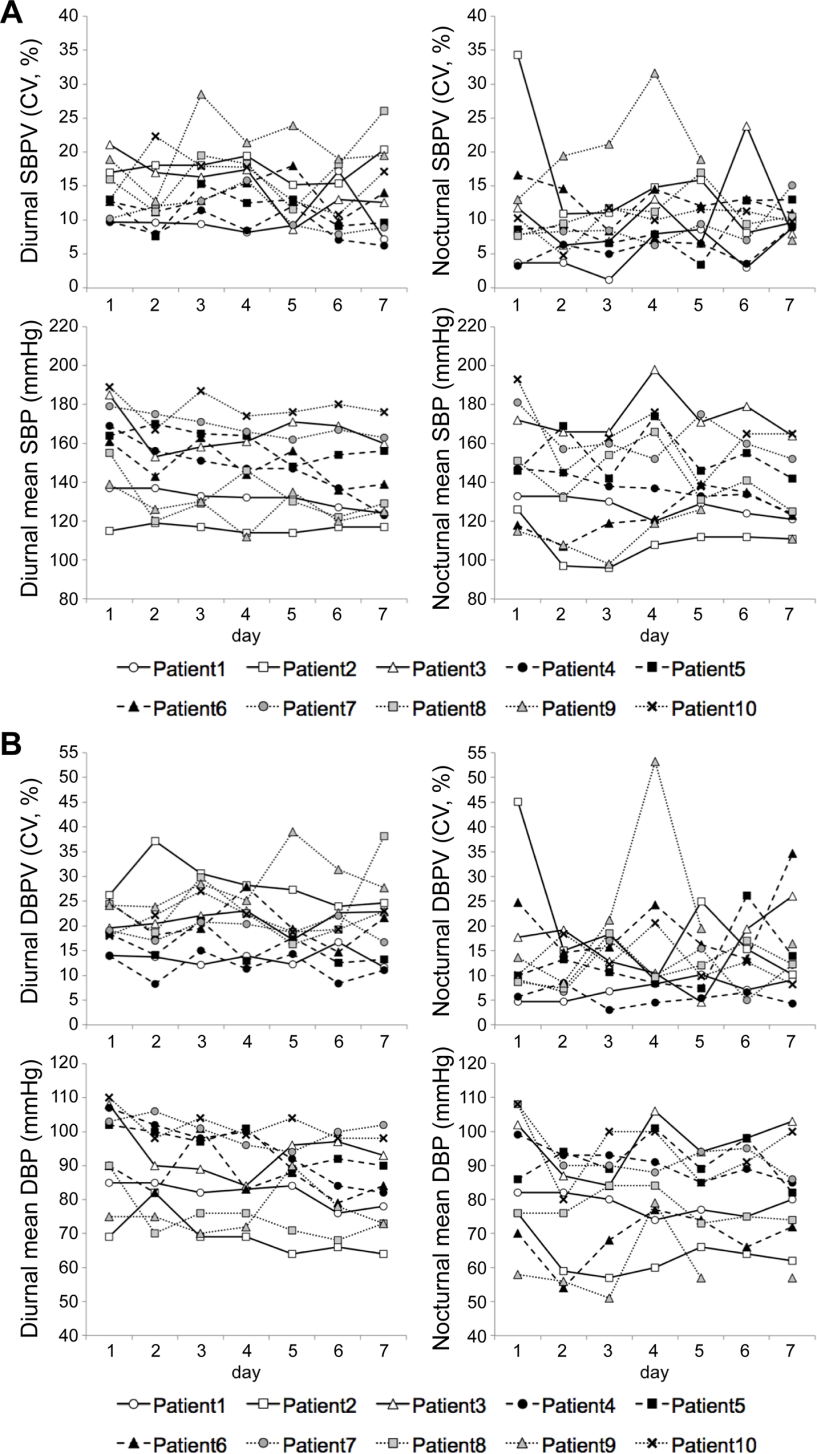
Changes in BP variability and mean BP value from day1 to day7. (A) CV for diurnal/nocturnal SBPV and diurnal/nocturnal mean SBP, (B) CV for diurnal/nocturnal DBPV and diurnal/nocturnal mean DBP. Mean SBP and DBP showed decreasing pattern from baseline for 7 days, however, both SBPV and DBPV showed greater day-by-day changes compared to those in mean BP values. 3SBPV, systolic blood pressure variability; DBPV, diastolic blood pressure variability.

After the 7-day treatment, median [QR] CV for diurnal SBP variability was changed from day 1 to day 7–13.0 [10.8 to 16.8] % to 13.3 [9.1 to 18.9] % (*P* = 0.959)—and median [IQR] change between days 1 and 7 was -0.3 [-3.2 to 2.9] % (Table 2). BP parameters other than the primary endpoint are shown in Table 3. In addition, CV for diurnal SBP variability varied greatly on a day-by-day basis for 7 days (Fig 2).

**Table 2:** Change in CV for diurnal SBP variability between days 1 and 7.

Variable	Day 1	Day 7	Difference between days 1 and 7	*P*-value
Diurnal SBP variability (CV, %)	13.0 (10.8 to 16.8)	13.3 (9.1 to 18.9)	-0.3 (-3.2 to 2.9)	0.959

Data are presented as medians (interquartile range: 25 to 75 percentiles).

CV, coefficient of variation; SBP, systolic blood pressure.

**Table 3:** Change in other BP parameters between days 1 and 7.

Variable	Day 1	Day 7	Difference between days 1 and 7
Diurnal mean SBP (mmHg)	163 (143 to 177)	134 (124 to 159)	-15 (-24 to -13)
Nocturnal SBP variability (CV, %)	9.5 (7.8 to 12.7)	9.7 (8.9 to 10.9)	1.2 (-5.2 to 4.9)
Nocturnal mean SBP (mmHg)	147 (128 to 167)	125 (122 to 150)	-14 (-25 to -5)
Diurnal DBP variability (CV, %)	19.4 (18.1 to 24.3)	22.3 (14.1 to 24.2)	-2.0 (-2.7 to 3.5)
Diurnal mean DBP (mmHg)	96 (86 to 106)	83 (74 to 92)	-10 (-14 to -5)
Nocturnal DBP variability (CV, %)	10.1 (8.8 to 16.7)	12.2 (9.4 to 15.8)	3.2 (-0.4 to 4.3)
Nocturnal mean DBP (mmHg)	84 (76 to 101)	81 (73 to 86)	-3 (-13 to -1)

Data are presented as medians (interquartile range: 25 to 75 percentiles).

CV, coefficient of variation; SBP, systolic blood pressure; DBP, diastolic blood pressure.

**Fig 3:**
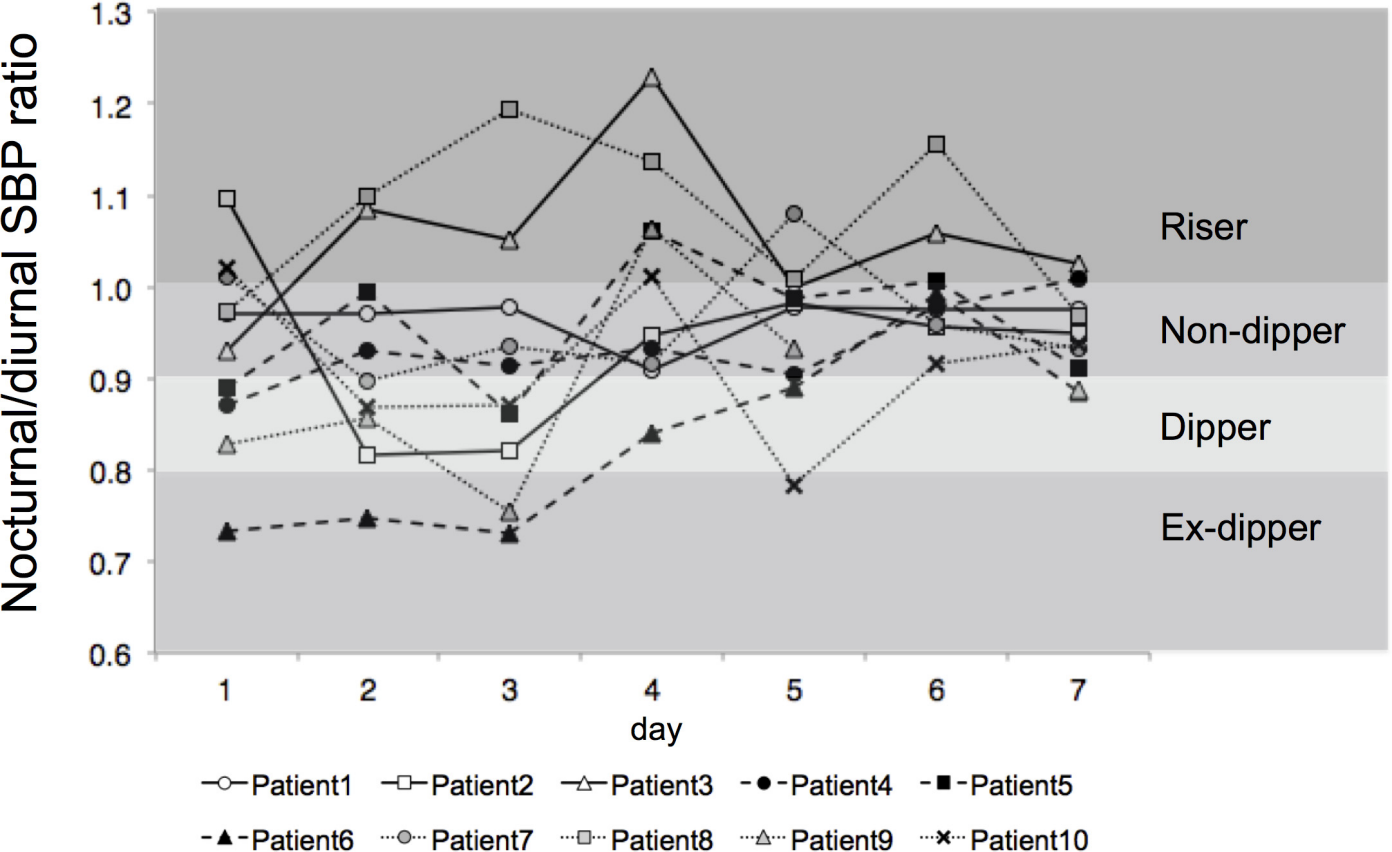
Change in circadian rhythm of BP from day1 to day7. Circadian rhythm of BP varied greatly on a day-by-day basis. The rate of dippers was not improved until day 7. Seven participants showed over three nocturnal/diurnal SBP ratio patterns.

With regard to circadian rhythm of BP, there was no increase in the proportion of those showing a “dipper” pattern after the 7-day treatment. Only one participant exhibited a similar pattern of circadian rhythm of BP (i.e., non-dipper) over the entire course of the study; 2 participants showed 2 distinct patterns; 7 showed 3 or more patterns; and 2 shifted between the “extreme-dipper” and the “riser” patterns over 2 consecutive days (Fig 3; Table 4).

**Table 4:** Day-by-day change in nocturnal/diurnal systolic blood pressure ratio.

	Day 1	Day 2	Day 3	Day 4	Day 5	Day 6	Day 7
Patient 1	Non-dipper	Non-dipper	Non-dipper	Non-dipper	Non-dipper	Non-dipper	Non-dipper
Patient 2	Riser	Dipper	Dipper	Non-dipper	Non-dipper	Non-dipper	Non-dipper
Patient 3	Non-dipper	Riser	Riser	Riser	Non-dipper	Riser	Riser
Patient 4	Dipper	Non-dipper	Non-dipper	Non-dipper	Non-dipper	Non-dipper	Riser
Patient 5	Dipper	Non-dipper	Dipper	Riser	Non-dipper	Riser	Non-dipper
Patient 6	Ex-dipper	Ex-dipper	Ex-dipper	Dipper	Dipper	Non-dipper	Dipper
Patient 7	Riser	Dipper	Non-dipper	Non-dipper	Riser	Non-dipper	Non-dipper
Patient 8	Non-dipper	Riser	Riser	Riser	Riser	Riser	Non-dipper
Patient 9	Dipper	Dipper	Ex-dipper	Riser	Non-dipper	ND	Dipper
Patient 10	Riser	Dipper	Dipper	Riser	Ex-dipper	Non-dipper	Non-dipper

Ex-dipper, extreme dipper; ND, not determined.

## Discussion

In this study, rigorous salt restriction during 7-day hospitalization led to decreases in mean BP values. Not only salt restriction but also hospitalization itself is reported to lead to reductions in sympathetic nerve activity and BP [14]. However, the major part of effect to reduce BP appears in about first 24 hours after admission [15]. For this reason, in 7-day treatment in this study, salt restriction was thought to be the main contributing factor in reducing mean BP values in participants. On the other hand, salt restriction during 7-day hospitalization did not lead to an improvement in short-term BP variability, and in fact led to a worsening of short-term BP variability in some participants.

Compared to an earlier study involving antihypertensive therapy [16], this study tended to show greater BP variability among participants. In this regard, the use of antihypertensive agents, even when less potent than calcium channel blockers, the most potent of all antihypertensive agents in reducing BP variability [17], may be more effective in reducing short-term BP variability compared to non-use of antihypertensive therapy.

Moreover, reduced baroreceptor reflex and increased sympathetic activity are counted among the factors contributing to short-term BP variability in individual patients [18,19]. While such BP variability is reported to be greater in diabetic patients [20], this is in line with the observation that not only the baroreceptor reflex is reduced but sympathetic activity is relatively increased in these patients [21,22]. The present study set out to explore the underlying causes of short-term BP variability in diabetic patients by assessing sympathetic activity in terms of LF/HF ratios. However, the only finding in this study was that sympathetic activity had no clear correlation with short-term BP variability (data not shown). On the other hand, while the baroreceptor reflex was not evaluated in this study, it has been reported in previous studies to be significantly decreased before the manifestation of dysautonomia in diabetic patients [23]. This suggests that the baroreceptor reflex may have contributed to high BP variability even in this study involving patients with relatively less advanced diabetic complications. Additionally, while aging is also known to be a risk factor for BP variability, no correlation was found between age and short-term BP variability in hypertensive patients with type 2 diabetes in this study. These observations suggest that increased short-term BP variability may represent another marker for hypertension that remains relatively constant in an individual, as it remains less affected by short-term salt restriction.

It is not clear yet whether the mean BP value or BP variability is more important. However, increased short-term BP variability appears to have far-reaching implications. For example, it may suggest that increased BP variability—remaining unchanged after marked reductions in mean BP values following a variety of therapeutic interventions—could lead to increases in exposure to asymptomatic hypotension, thus accounting for the failure of excessive BP lowering to produce desired effects [24].

Furthermore, diabetic patients are often shown to exhibit a “non-dipper” pattern of circadian rhythm of BP [25], which is thought to be due to decreases in nocturnal sodium excretion, thus causing salt-sensitive hypertension. Given that diabetic patients often exhibit increased salt sensitivity [26], it is likely that almost all patients in this study had salt-sensitive hypertension, except for the 2 patients who had a lower salt intake after admission and in whom such evaluation proved difficult.

Contrary to earlier reports showing that salt restriction leads to a shift in circadian rhythm of BP from a “non-dipper” to a “dipper” pattern [27]—and that the use of diuretics leads to a similar shift [28]—our study demonstrated that circadian rhythm of BP varied greatly from one day to the next and that circadian rhythm of BP was not improved with salt restriction or even worsened in some participants. While ABPM is thought to cause sleep disorders soon after its use, thus adversely affecting circadian rhythm of BP, virtually no participants complained of insomnia during interviews in this study. Indeed, whether or not ABPM affected it, circadian rhythm of BP, did not improve after salt restriction compared to baseline in these participants.

Our study results suggest that circadian rhythm of BP varies greatly, thereby likely accounting for patterns that may be less reproducible and less well characterized in hypertensive patients with type 2 diabetes and that highly variable day-by-day changes in circadian rhythm of BP may remain a risk factor even after risk reduction with salt restriction.

**Fig 4:**
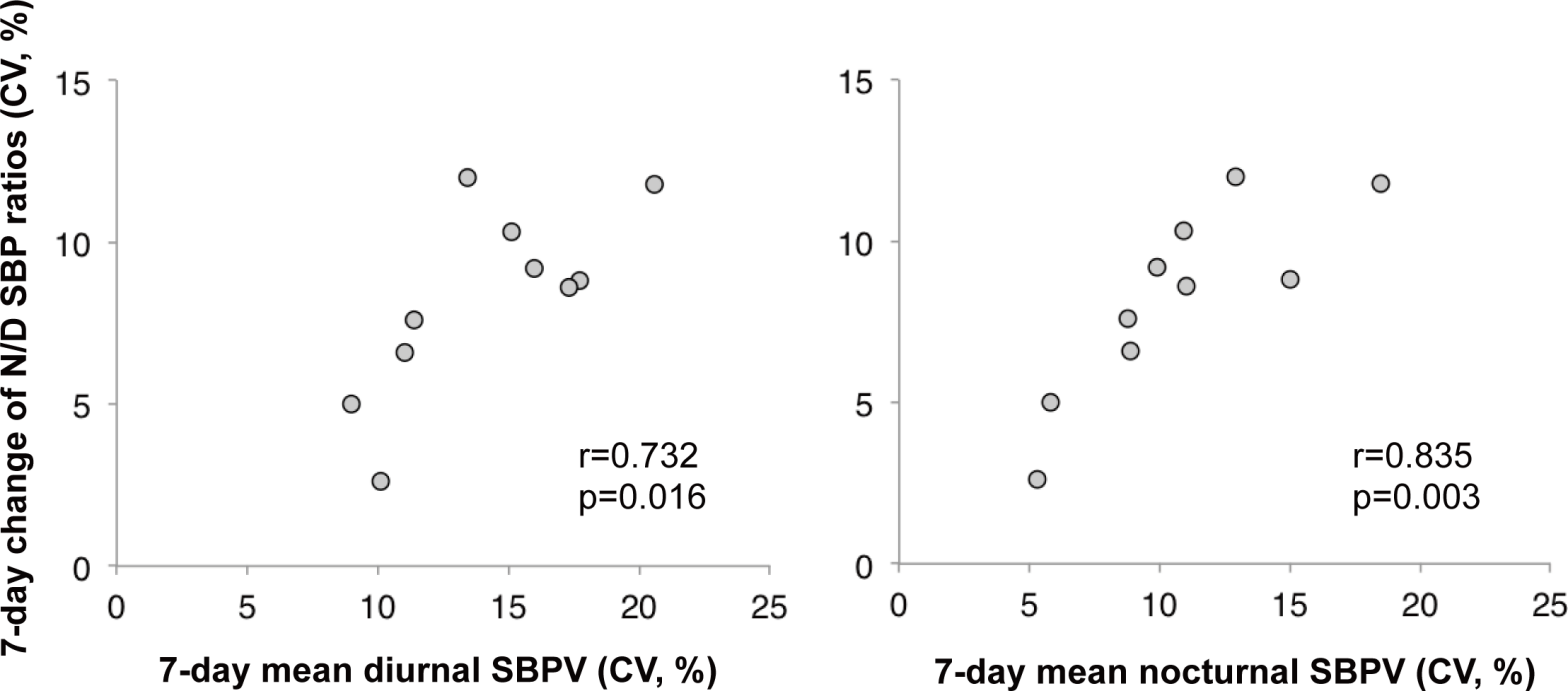
Correlation between 7-day change in N/D SBP ratio and 7-day mean SBPV. Increased diurnal and nocturnal SBPV is correlated with day-by-day changes in nocturnal/diurnal SBP ratio. 3N/D SBP ratio, nocturnal/diurnal systolic blood pressure ratio; SBPV, systolic blood pressure variability.

These highly variable day-by-day changes in circadian rhythm of BP may be explained by the observed 7-day changes in circadian rhythm of BP (CV for 7-day change of nocturnal/diurnal SBP ratios) and 7-day mean short-term SBP variability (CV) being correlated with both diurnal and nocturnal SBP variability (Fig 4). Thus, circadian rhythm of BP may be influenced by increased short-term BP variability and may not have been captured accurately in such patients.

This study has the following limitations: First, no control group, such as hypertensive patients without type 2 diabetes, was in place and the sample size was small, due to difficulties involving hypertensive patients not receiving antihypertensive agents and not being able to obtain informed consent from patients to undergo both ABPM and CGM over a 7-day period. Second, the study may have been associated with selection bias as it was not a randomized study. Third, the study was not powered to detect decreases in BPV with the range of BPV decrease predetermined for detection; the study may have been associated with type II errors due to not significantly different results being produced. Fourth, the study was not designed as a long-term study and patients were followed up for one week only. Although it is generally accepted that long-term salt restriction leads to reductions in BP levels and variability, one study reported that salt restriction over 36 months did not decrease visit-to-visit BP variability [29]. Fifth, short-term glycemic control status may have affected BP parameters in some way, given the STOP-NIDDM study finding that long-term glycemic control leads to reductions in the incidence of hypertension [30]. Moreover, hyperglycemia persisting over the long term, or high glycemic variability, may have been implicated in BP variability as a background factor. Despite these limitations, however, we believe we have provided new and important insights into BP variability and circadian rhythm of BP in hypertensive patients with type 2 diabetes.

## Conclusions

Salt restriction during 7-day hospitalization led to a -0.3 [-3.2 to 2.9] (median [IQR]) % change from baseline in CV for diurnal SBP variability in 10 hypertensive patients with type 2 diabetes not receiving antihypertensive agents. In addition, short-term BP variability showed greater day-by-day changes compared to mean BP values. Furthermore, increased SBP variability is associated with greater day-by-day changes in circadian rhythm of BP.

## Supporting Information

S1 File Study protocol English version. (PDF)

S2 File Study protocol Original Language version. (PDF)

S3 File Trend checklist. (PDF)
