# Supplementary material for: Effect of One-Week Salt Restriction on Blood Pressure Variability in Hypertensive Patients with Type 2 Diabetes
Source: PLoS One. 2016 Jan 5;11(1):e0144921. doi: 10.1371/journal.pone.0144921 (PMC4701465; doi:10.1371/journal.pone.0144921)
Supplement: S1 File — (PDF) [file pone.0144921.s001.pdf]

THE JIKEI UNIVERSITY
SCHOOL OF MEDICINE

3-25-8 Nishi-shinbashi, Minato-ku, Tokyo 105-8461, Japan
Tel : 81-3-3433-1111 Fax : 81-3-3433-6128
OFFICE OF THE PRESIDENT

*May 8, 2015*

TO WHOM IT MAY CONCERN:

Field of Study : Examination of the effect of Salt Restriction on Blood Pressure Variability in Hypertensive Patients with Type 2 Diabetes

Registration Number : 25-196 (7331)

Date : 2013/10/07

From(mo/yr) : 2013/10/24

This is to certify that the above field of study has been approved by the Ethics Committee of The Jikei University School of Medicine for Biomedical Research.

Ethics Committee ( II ) Chairperson :

Kimiharu Iwadate

Kimiharu Iwadate, MD., Ph.D.

Head Official of Organizational Operations of the Ethics Committee :

Senya Matsufuji

Senya Matsufuji, MD., Ph.D.

**Research Protocol**

*Hiroyuki Iuchi*, MD
Division of Diabetes, Metabolism and Endocrinology
Department of Internal Medicine
Jikei University School of Medicine, Tokyo, Japan

# Effect of Salt Restriction on Blood Pressure and Blood Pressure Variability

## 1. Background to the Proposed Research

It is reported in recent years that not only absolute blood pressure (BP) values but also blood pressure variability (BPV) influence the occurrence of cardiovascular (CV) events, independently of BP values,[1] and BPV is becoming the focus of attention. Furthermore, BPV is shown to be particularly significant in patients with diabetes mellitus, with a sub-analysis of the ADVANCE trial showing that BPV is among the factors that contribute to worsening of both microangiopathy and macroangiopathy in diabetic patients.[2]

BPV, as reported in these studies, represents visit-to-visit BPV or that observed in the clinic BP measurement during outpatient visits made on an approximately monthly basis. BPV as assessed by the clinic BP measurement is now assumed to be accounted for not only by the status of BPV in each individual patient but by other environmental factors, which include the patient's antihypertensive drug compliance, "white coat hypertension" involving another measurer than the patient himself/herself, i.e., a healthcare provider in the clinic, seasonal BP variations, and the patient's dietary habit. Again, short-term BPV can be variously expressed as day-by-day BPV as assessed by home BP monitoring (HBPM) or short-term BPV as assessed at 30-minute or 1-hour intervals using 24-hour ambulatory BP monitoring (ABPM); while it is recently suggested that either of these short-term BPV indices is associated with worsening of diabetic complications and increased risk of mortality in diabetic patients, how they may be correlated remain yet to be elucidated in detail.

We found earlier that day-by-day BPV and short-term BPV are correlated in diabetic patients with hypertension and reported on this correlation at the 2013 Annual Meeting of the Japanese Society of Hypertension. Of note, our study findings also suggested that increases in short-term BPV may be associated with increases not only in day-by-day BPV but also in long-term visit-to-visit BPV.

While the drugs reported to suppress long-term visit-to-visit BPV as assessed by the clinic BP measurement include calcium channel blockers (CCBs) and diuretics, very few studies evaluated these agents for their ability to suppress day-by-day or short-term BPV. Again, while lifestyle modification, particularly salt restriction, should precede any pharmacological intervention as part

of any attempt to reduce BP levels in patients requiring intervention for BP control, to date, very few studies have evaluated the effect of salt restriction on day-by-day BPV, short-term BPV, or long-term BPV. This study was therefore designed to evaluate salt restriction for its effect on day-by-day BPV and short-term BPV, respectively.

## 2. Objective

The primary objective of this study is to evaluate the influence of salt restriction on day-by-day BPV and short-term BPV in eligible patients with hypertension admitted for rigorous salt restriction. The secondary objectives include analysis of correlation between glucose variability as assessed by 24-hour continuous glucose monitoring (CGM) and BPV; and assessment of BPV based on changes in autonomic nerve activity as assessed by LF/HF ratio, heart rate, urinary sodium (Na) excretion, body weight, and renin-aldosterone values.

## 3. Methods

① *Study design*

This is designed as an observational study.

② *Study protocol*

- All consenting, eligible patients will be admitted for 7 nights/8 days to undergo salt restriction with their salt intake limited to 6 g/day.
- All patients will be subjected to simultaneous BP and glucose assessment using ABPM (TM-2431; A & D Technology Inc) and CGM (iPro 2; Medtronic Inc) for 7 consecutive days.

③ *Study schedule*

The study schedule is projected as shown below.

| Item/variable | Before admission | Hospital admission | | |
|---|---|---|---|---|
| | | Day 1 | Day 2 | Days 3 to 7 |
| Written informed consent | ○ | | | |
| Patient background survey/enrollment | ○ | | | |
| BP measurement | | ○ | ○ | ○ |
| Body weight measurement | | ○ | ○ | ○ |
| Hematology/blood chemistry | | | ○ | |
| Urinalysis | | ○ | ○ | ○ |
| ABPM | | ○ | ○ | ○ |
| CGM | | ○ | ○ | ○ |
| Pulse monitoring | | ○ | ○ | ○ |

BP, blood pressure; ABPM, ambulatory blood pressure monitoring; CGM, continuous glucose monitoring

*Duration of hospital admission*: 7 nights/8 days from Monday through next Monday

*ABPM*: Each patient is to wear an ABPM device on the day of admission and to continue to wear it for 7 consecutive days, and the device is to be collected on the day of his/her discharge.
*CGM*: Each patient is to put on a CGM device on the day of admission and to continue to wear the device for 7 consecutive days for glucose monitoring, and the device is to be collected on the day of his/her discharge.
*Pulse monitoring*: Each patient is to put on a pulse monitor (RS800 CX; Polar Electro) on the day of admission and to continue to wear the device for 7 consecutive days for pulse monitoring, and the device is to be collected on the day of his/her discharge.
*Blood sampling*: Blood to be sampled from each patient once on the day after admission (day 2 of admission).
*Urinalysis*: Urinalysis is to be performed for each patient using first urine samples taken from day 2 of admission through the day of his/her discharge.
*BP and body weight*: To be measured for each patient every morning during admission.

④ *Assessment/variables to be assessed*
*ABPM*: Systolic BP (SBP) and diastolic BP (DBP) are to be measured for their mean values, their standard deviations (SDs) and coefficient of variation (CV) over 24 hours, during daytime and during nighttime, to allow analysis of BPV for each period.
*CGM*: Blood glucose is to be monitored in each patient for SDs of 24-hour glucose, CV, mean amplitude of glycemic excursions (MAGE), and AUC (postprandial 3-hour value).
*Parameters to be evaluated from blood samples*: Na, K, fasting plasma glucose (FPG), serum C-peptide, HbA1c, triglycerides (TG), high-density lipoprotein cholesterol (HDL-C), renin activity, and aldosterone concentration
*Parameters to be evaluated through urinalysis*: general urine parameters, urinary Na, urinary K, urinary creatinine (Cr), and urinary albumin

⑤ *Study endpoints*
Primary endpoint: Decreases in BP
Secondary endpoint: Decreases in urinary sodium (Na) excretion

⑥ *Informed consent protocol*
The study investigator is to explain to each patient the clinical examinations he/she is to undergo during the course of the study as well as the risk and benefit of these examinations during consultations in the outpatient clinic.

**4. Study subjects**

① *Inclusion criteria*

Type 2 diabetic patients with hypertension 20 years of age or older are judged eligible for participation in the study if they are scheduled to be admitted for diabetes education; if they are not receiving antihypertensive agents; and if they gave informed consent to participate in the study.

② *Exclusion criteria*

Patients are judged ineligible for participation in the study if they are already receiving antihypertensive agents; if they are being treated with diuretics; if they are found to have serious hypertension (grade III or higher) or symptomatic hypertension, serious renal impairment (serum Cr ≥ 2.5 mg/dL), serious hepatic dysfunction (AST/ALT values, ≥ 3 times upper limit of normal; hepatic cirrhosis), serious infection, serious injury, serious ketosis, diabetic coma, or insulin dependence; if they are scheduled for surgery or they had just undergone surgery; if they are pregnant or potentially pregnant; and if they are judged by the study investigator to be ineligible for the study.

For all patients who do not give informed consent to participate in the study, appropriate care will be provided to ensure that their decision not to participate in the study will not adversely affect their health and well-being.

**5. Target number of patients and duration of the study**

① *Duration of the study*: from October 2013 to September 2014 (for a 12-month period)

② *Target number of patients*: 20 patients

**6. Study site**

Katsushika Medical Center, Jikei University School of Medicine, Tokyo, Japan

Division of Diabetes, Metabolism and Endocrinology

Outpatient Clinic: Block D

In-patient wards: Mainly 9B, 9A, 8B, 8A, and 6A

**7. Safety Considerations (anticipated adverse events and their countermeasures)**

① *Anticipated adverse events*

- Those associated with ABPM: insomnia

② *Measures to be taken against cases of this anticipated adverse event (i.e., insomnia)*

- Patients experiencing this event are to be given a benzodiazepine sleep medication.
- The principal investigator/co-investigator is to withdraw all patients not responding to the

benzodiazepine sleep medication from the study and provide appropriate care.

③ *Criteria for patient withdrawal from the study*

If a patient has met any of the following criteria, he/she is to be withdrawn from the study. All patients withdrawn from the study are to be recorded on their case report forms as such, with the reasons for their withdrawal specified:

- If he/she developed associated symptoms or untoward symptoms and was judged unable to continue the study;
- If he/she showed evidence of an underlying disease or a concomitant disease worsening and was judged unable to continue the study;
- If he/she or his/her agent expressed a desire to discontinue the study;
- If he/she was found to have failed to meet the inclusion/exclusion criteria for the study;
- If he/she was found to be using any drug forbidden in the study;
- If he/she had any reason for his/her withdrawal from the study (e.g., withdrawal of informed consent, desire to discontinue the study, unavailability, or change of hospital or address);
- If his/her case constituted a violation of the study protocol for other reasons; and
- If he/she was judged by the study investigator to require study withdrawal for health reasons.

**8. The risk and benefit for the patients participating in the study**

*Benefit*: By participating in the study, each patient will have an opportunity to have their BP values evaluated through a rigorous protocol as well as to choose antihypertensive drugs for improved BP control, thereby avoiding worsening of his/her prognosis.

*Risk*: By participating in the study, each patient may be hospitalized for a prolonged period which may result in an additional consultation burden.

**9. Other common treatments to be made available to the patients**

Each patient will be offered instructions on salt restriction as part of his/her antihypertensive therapy as well as appropriate antihypertensive agents, both of which will be offered as part of their antihypertensive therapy, irrespective of their eventual decision to/not to participate in the study.

**10. Cost considerations**

- As deemed necessary for any primary/underlying disease present in each patient, a range of clinical examinations, procedures, and treatments covered by insurance will be implemented during the course of the study.
- As all study procedures to be implemented will be covered by insurance, participation in the

study will result in no additional cost burden on each patient, beyond costs for admission for diabetes care and pharmacological treatments given.

- Therefore, each study participant is expected to cover only healthcare costs which will be due him/her within the national health insurance system.

**11. Handling of clinical samples and data**

① *Handling of clinical data during the study*

All "personally identifiable information" is to be stored in an electronic file password-protected for access only in laboratories designated by the study organizer, i.e., Division of Diabetes, Metabolism and Endocrinology, Jikei University School of Medicine, and for internal use/reference only.

② *Handling of clinical data after the study*

In analysis of clinical data, the study investigators are to create a separate database, from which all "personally identifiable information", such as patient names or addresses, is removed.

**12. Contact details for emergency calls (telephone number and contact details)**

All queries or requests for consultation regarding the study should be addressed to:

*Hiroyuki Iuchi*, MD

c/o Division of Diabetes, Metabolism and Endocrinology, Katsushika Medical Center, Jikei University School of Medicine, Tokyo, Japan

Address: 6-41-2 Aoto, Katsushika-ku, Tokyo 125-8506, Japan

Phone: +81 (0)3 3603 211 Ext. 5979 Fax: +81 (0)3 3838 9943 (9944/9945)

**13. Study investigators/facilities**

*Study investigators/facilities*

*Hiroyuki Iuchi*, MD (Division of Diabetes, Metabolism and Endocrinology, Katsushika Medical Center, Jikei University School of Medicine, Tokyo, Japan)

*Kennosuke Ohashi*, MD (Division of Diabetes, Metabolism and Endocrinology, Katsushika Medical Center, Jikei University School of Medicine, Tokyo, Japan)

*Sho Ishizawa*, MD (Division of Diabetes, Metabolism and Endocrinology, Katsushika Medical Center, Jikei University School of Medicine, Tokyo, Japan)

*Tamotsu Yokota*, MD (Division of Diabetes, Metabolism and Endocrinology, Katsushika Medical Center, Jikei University School of Medicine, Tokyo, Japan)

*Principal study investigator*

*Hiroyuki Iuchi*, MD (Division of Diabetes, Metabolism and Endocrinology, Katsushika Medical Center, Jikei University School of Medicine, Tokyo, Japan)

**14. Research costs**

Sundry expenses associated with the study are to be covered by the research budget allocated to Division of Diabetes, Metabolism and Endocrinology, Katsushika Medical Center, Jikei University School of Medicine, and are not to be covered by public or corporate research grants or funds.

**15. Conflicts of interest**

Being a self-funded research project, this study is associated with no such conflict of interest as would interfere with the transparency or validity of the study being conducted.

**16. Study registration with a public research database (as clinical research involving invasive approaches)**

This study will be registered with a public research database run in line with the Guidelines for Clinical Research set forth by the National University Hospital Directors Council.

**17. Measures to be taken for protection of human rights and personal privacy**

- All personal information for the study participants will be handled in line with the "Jikei University Regulations for Protection of Personal Information", the "By-laws for Obtainment, Utilization and Provision for Third-party Use of Patient Personal Information" and the "Ethical Guidelines for Clinical Studies."
- None of the personal information obtained on the study participants during the course of the study will be made available for use by a third party.
- Requests for disclosure of personal information for any study participant, complaints or queries regarding the study should be addressed to: Consultation Counter for Protection of Personal Information, Katsushika Medical Center at +81 (0)3 5400 1272 Ext. 2590 (available from 9:00AM to 5:00PM, except on "non-consultation" days)
- Results from the study are scheduled for reporting at relevant professional society meetings, with all personally identifiable information removed from the results.

**18. References**

1. Rothwell PM, Howard SC, Dolan E, et al. Prognostic significance of visit-to-visit variability, maximum systolic blood pressure, and episodic hypertension. *Lancet* 2010;375:895-905.
2. Hata J, Arima H, Rothwell PM, et al. Effects of visit-to-visit variability in systolic blood pressure on macrovascular and microvascular complications in patients with type 2 diabetes mellitus. The ADVANCE Trial. *Circulation* 2013;128:1325-1334.
