# Supplementary material for: Effect of One-Week Salt Restriction on Blood Pressure Variability in Hypertensive Patients with Type 2 Diabetes
Source: PLoS One. 2016 Jan 5;11(1):e0144921. doi: 10.1371/journal.pone.0144921 (PMC4701465; doi:10.1371/journal.pone.0144921)
Supplement: S2 File — (PDF) [file pone.0144921.s002.pdf]

様式2

平成26年 3月 11日

倫理委員会審査結果通知書

所属　葛飾医療センター糖尿病・代謝・内分泌内科
申請者　横田太持 診療部長　殿

東京慈恵会医科大学
学長　松藤千弥

受付番号　25-238　7373

研究課題名
高血圧合併2型糖尿病患者における血圧変動の検討

研究代表者　井内裕之　診療医員

上記申請を倫理委員会で審査した結果、下記のように判定したので通知します。

審査年月日　平成26年3月3日

1. 認める　　2. 認めない　　3. 申請を要しない　　4. 修正を要する

＊介入を伴う臨床研究で侵襲性を有する研究を実施する場合,研究開始前に公的なデータベースへの研究登録を行うこと（臨床研究に関する倫理指針第1.3用語の定義.(1)①及び②,第2.2.(5)）

＊各附属病院が実施施設の場合、臨床研究審査委員会の審査を通過しないと研究は実施できません

研究実施計画書

糖尿病・代謝・内分泌内科　井内裕之

「減塩による血圧値、血圧変動への影響の検討」

１．研究の経緯・背景

近年、血圧値の絶対値だけでなく、血圧値の変動が血圧値と独立して心血管イベントに影響することが報告され [1]、血圧変動に注目が集まっている．また糖尿病患者では血圧変動が大きくなることが判明しており、さらに糖尿病患者における血圧変動は、細小血管障害、大血管障害共に増悪させる因子であることが ADVANCE 試験のサブ解析で判明した．[2]

これらの報告の血圧変動は、約１ヶ月毎の外来時血圧変動である．外来時血圧変動は個人の血圧の変動以外にも、内服コンプライアンス、測定者の違いによる白衣高血圧、季節変動、食事による影響などの環境因子が多く関わっていると考えられている．本来の個人の血圧変動は、より短期の血圧変動で検討する必要性がある．短期の血圧変動としては、日ごとの血圧変動である、血圧日差変動や、24 時間血圧測定(ambulatory blood pressure monitorning:ABPM)で測定される、30 分～1 時間毎の血圧短期変動が挙げられる．血圧日差変動、血圧短期変動も、糖尿病患者の合併症増悪、死亡リスクに繋がる報告がみられるようになってきているが、両者の関係性については明らかになっていない.

我々は高血圧合併糖尿病患者において血圧日差変動と血圧短期変動が関連することを確認し、2013 年高血圧学会にて発表した．この検討により血圧変動は血圧短期変動が増大すると、血圧日差変動が増大し、さらに外来時血圧変動の増大にも繋がることが予想された.

外来時血圧変動を抑制するものとして、薬剤では Ca 拮抗薬、利尿剤が挙げられているが、それらの薬剤が血圧日差変動、血圧短期変動を抑えるかの検討は乏しい．また本来血圧値を抑制するには生活習慣の改善が先に立つべきであり、減塩を行うことは必須である．現時点で減塩による血圧変動を統計学的に検討したものは、外来時血圧変動、血圧日差変動、血圧短期変動のいずれにおいてもない.

本研究にて減塩の血圧日差変動、血圧短期変動に及ぼす影響を検討する.

２．目的

入院での厳格な減塩を行い、減塩により血圧値、各血圧変動に与える影響を検討する．また副次的項目として、24 時間持続血糖測定(continuous glucose monitoring:CGM)により測定した血糖変動と血圧変動の関連の解析を行う．その他、LF/HF による自律神経変動、心拍変動、尿中 Na 排泄量の変動、体重の変動、レニン・アルドステロン値での血圧変動の検討を行う.

３．方法

①試験デザイン：観察研究

②試験方法

- 7 泊 8 日の入院にて 6g の塩分制限を行う.
- 7 日連続で ABPM（TM-2431：A&D 社)、CGM（iPro2：メドトロニック社）を同時に施行する.

②試験スケジュール

試験スケジュールの概要を以下にまとめる。

観察・検査項目	入院前	入院<br>第１病日	入院<br>第２病日	入院<br>第３～７病日
文書での同意取得	○			
患者背景調査・登録	○			
血圧測定		○	○	○
体重測定		○	○	○
一般血液・生化学検査			○	
尿検査		○	○	○
ABPM		○	○	○
CGM		○	○	○
脈拍測定器		○	○	○

入院：月～翌月曜日の 7 泊 8 日

ABPM：入院日に装着し、退院日に回収。連続 7 日間使用。

CGM：入院日に装着し、退院日に回収。連続 7 日間使用。

脈拍測定器（polar 社 RS800CX）：入院日に装着し、退院日に回収。連続 7 日間使用。

採血：入院翌日に 1 回施行。

尿検査：入院第２病日から退院日まで毎朝第一尿を施行。畜尿も併せて実施.

血圧・体重測定：入院中連日朝に施行。

④評価方法・項目

ABPM：

SBP、DBP、脈圧を Mean（平均値）、SD（標準偏差）、CV（変動係数）を 24 時間、日中、夜間に分けて計測. また血圧日内変動を解析.

CGM：

SD（標準偏差）、CV（変動係数）、Mean Amplitude of Glycemic Excursions（以下 MAGE）、AUC（食後 3 時間の面積）を計測。

採血項目：

Na、K、空腹時血糖、血中 C ペプチド、HbA1c、TG、LDL-C、HDL-C、レニン活性、アルドステロン

尿検査項目：

尿一般、尿中 Na、尿中 K、尿中 Cr、尿中 C ペプチド、尿中アルブミン

⑤エンドポイント

プライマリー・エンドポイント：血圧値の低下

セカンダリー・エンドポイント：尿中 Na 排泄量の低下

⑥インフォームド・コンセントの方法

担当医師が外来にて、本試験の検査方法、得られる利益、不利益について説明する。

４．対象（選択基準など）

①対象患者

対象：教育入院予定の高血圧合併 2 型糖尿病患者

年齢：20 歳以上

内服薬：降圧薬を内服していない患者

同意：試験参画に同意が得られた患者

②除外例(基準)

・降圧薬をすでに内服している患者
・利尿薬を使用している患者
・Ⅲ度以上の重篤な高血圧を有する、または高血圧による症状を有する患者
・重篤な腎機能障害を有する患者（血清 Cr ≧2.5mg/dL）
・重篤な肝機能障害を有する患者（AST、ALT が正常上限の 3 倍以上、肝硬変）
・重症感染症、手術前後、重篤な外傷のある患者
・重症ケトーシス、糖尿病性昏睡、インスリン依存型糖尿病の患者
・妊婦及び妊娠している可能性のある患者
・その他、主治医が不適と判断した患者

なお試験参加に同意しなかった患者に対しては、患者の不利益になることのないよう、必要に応じた適切な処置を行う。

５．目標症例数及び予定期間

① 研究期間：２０１３年１０月～２０１４年９月（１２ヶ月）

② 症例数：２０例

６．実施場所

東京慈恵会医科大学　葛飾医療センター

糖尿病・代謝・内分泌内科

外来：D ブロック

入院病棟：9B、9A、8B、8A、6A 病等

７．安全性の確保について（予想される有害事象と対策）

①予想される有害事象

・ABPM による有害事象

不眠

②有害事象が発生した場合の対処方法

ベンゾジアゼピン系睡眠導入剤の処方.

ベンゾジアゼピン系睡眠導入剤にて症状の改善が認められない場合は、試験責任医師、又は分担医師は直ちに試験中止とし、適切な処置を行う。

③中止基準

以下のような場合には試験を中止する。試験を中止した場合は、その理由を明らかにし

て記入用紙に記載する。

- 重篤な随伴症状、または偶発症が発現し、継続が不可能と認めた場合
- 原疾患、または合併症の症状が悪化し、継続が不適切と判断された場合
- 患者または代理人より中止の申し出があった場合
- 選択基準違反、除外基準抵触が判明した場合
- 使用禁止薬の使用
- 患者側の理由（同意の撤回、試験中止の申し入れ、多忙、転院、転居）
- その他の研究計画書違反が判明した場合
- その他、研究担当医師が医学的判断により中止の必要性を認めた場合

８．研究に参加する利益と不利益について

利益：被験者にとって厳格な血圧値の評価ができ、より良い血圧コントロールを行うための降圧薬が選択でき、予後悪化を回避できる可能性がある。

不利益：入院期間の増加が見込まれ、診療時間の負担が増加する。

９．他の一般的な治療方法

高血圧治療として減塩指導を受け、降圧薬が使用できる。患者が研究に参加しない場合もこれらを適切に使用し、治療を行う。

10. 費用について

- 原疾患、他の合併症に対する治療に準じ試験参加中の患者に対する検査、処置、治療は健康保険の範囲内で行われる。
- 保険範囲内で行われるため、試験参加に伴って糖尿病治療入院費、薬剤以外に関して特別に費用負担が増加することはない。
- 発生する費用は健康保険制度に基づいて患者は自己負担の部分を支払うことになる。

11. 検体やデータの取扱いについて

①研究期間中の取扱い

患者本人を特定できる情報は、パスワードブロックされたファイルで管理することとし、当科の指定された研究室内で閲覧することとし、これらの情報は保存場所から一切持ち出さないこととする。

②研究終了後の取扱い

研究者がデータ解析を開始する際は、本人を特定できる姓名、住所等の情報は削除したデータベースを作成し使用する。

12. 緊急時の連絡先（電話番号・担当者氏名）

研究に関する質問、相談の連絡先

部署：東京慈恵会医科大学　葛飾医療センター　糖尿病・代謝・内分泌内科

担当医師：井内　裕之

住所：〒125-8506 東京都葛飾区青戸 6-41-2

電話番号：03-3603-2111（内線：5979）

Fax：03-3838-9943～9945

13. 研究組織について

参画施設・医師

井内　裕之　（東京慈恵会医科大学　葛飾医療センター　糖尿病・代謝・内分泌内科）
大橋　謙之亮（東京慈恵会医科大学　葛飾医療センター　糖尿病・代謝・内分泌内科）
石澤　将　　（東京慈恵会医科大学　葛飾医療センター　糖尿病・代謝・内分泌内科）
横田　太持　（東京慈恵会医科大学　葛飾医療センター　糖尿病・代謝・内分泌内科）

試験責任医師

井内　裕之　（東京慈恵会医科大学　葛飾医療センター　糖尿病・代謝・内分泌内科）

14. 研究費について

本研究のうち、諸経費は葛飾医療センター糖尿病・代謝・内分泌内科の研究費より支出し、研究費補助金、企業などからの委託はなし。

15. 利益相反について

本研究は、企業や団体との利害関係はないため、利害の衝突によって研究の透明性や信頼性が損なわれるような状況は生じない。

16. 公的データベースへの登録（侵襲性を有する臨床研究）

本研究は国立大学附属病院長会議の臨床研究に関する倫理指針に対応するデータベース登録を行う。

17. X．人権・プライバシー保護に関する配慮

・本研究に係わる研究対象者の個人情報は、「学校法人慈恵大学　個人情報保護に関する規定」、「個人情報の取得・利用ならびに第三者提供に関する細則」及び「臨床研究に関する倫理指針」を遵守して取り扱う。

・　本研究に係わる研究対象者の個人情報は第三者へ提供しない。

・　研究対象者の個人情報の開示等の請求、苦情及び問い合わせ先は研究責任者の他次の通りである。

附属病院個人情報保護相談窓口
葛飾医療センター：03-5400-1272　内線：2590
午前9時～午後5時/休診日を除く

・　本研究の成果を論文、学会にて報告する予定があり、その際は被験者の氏名やイニシャルなど個人を特定できる情報がわからないよう配慮する。

18. 参考文献

１）*Lancet* 375, 895–905 (2010).
２）*Circulation. published online August*
